# Editorial Comment to Spontaneous regression of multiple pulmonary metastases accompanied by normalization of serum immune markers following cytoreductive nephrectomy in a patient with clear‐cell renal cell carcinoma

**DOI:** 10.1002/iju5.12256

**Published:** 2021-01-20

**Authors:** Kojiro Ohba

**Affiliations:** ^1^ Department of Urology and Renal Transplantation Nagasaki University Hospital Nagasaki City Nagasaki Japan

We rarely encounter the spontaneous regression of metastatic renal cell carcinoma (RCC) in the clinic which it occurs in less than 1% of all cases of RCC.^1^ A number of these cases has been observed with synchronous lung metastases, and regression has been occurred after cytoreductive nephrectomy (CN). A report presenting a case of spontaneous regression of RCC speculated that this phenomenon might be associated with activation of the immune system,^2^ while several other reports hypothesized that spontaneous regression is associated with the immune microenvironment. Rini and Campbell reported that CN led to decreased myeloid‐derived suppressor cells and regulatory T cells which suppress the host immune system, and decrease immunosuppressive cytokines such as interleukin‐10 and TGF‐β.^3^ Furthermore, CN may also decrease some growth factors and angiogenetic factors that are produced by RCC. From these hypothetical mechanisms evoked by CN, it may be reasoned that an enhanced antitumor response surpasses the immunosuppressive tumor microenvironment, resulting in spontaneous regression of metastases. However, the mechanisms of this spontaneous regression still remain unclear. Furthermore, the role of CN has been questioned by recent prospective studies, in spite of some cases of spontaneous regression. For example, serum laboratory markers such as C‐reactive protein and neutrophil‐to‐lymphocyte ratio are considered to be indicators of tumor aggressiveness, and these markers have been used as prognostic factors in patients who are unlikely to benefit from CN.

However, the findings of this current report contradict previous studies, and thus we have no clear idea as to which cases can be expected to undergo spontaneous regression of metastases following CN. While we reconfirmed that CN has an important role, further studies are required to identify markers that can predict the benefit of CN before surgery.

One limitation of this study was the lack of histopathological data for the lung nodule, and there was no evidence that the nodules were lung metastases. Furthermore, the spontaneous regression was not a cure. While the period from CN to spontaneous regression ranges from a few days to 1 year,^4^ many cases recur. Regarding the period from spontaneous regression to recurrence, one report stated that 20% recurred within 1 year, 17% recurred in 1–2 years, 23% in 2–5 years, and 40% in over 5 years.^5^ Hence, the current case requires long‐term follow‐up.

## Conflict of interest

The author declares no conflict of interest.
